# Endoscopic Ultrasound in Squamous Cell Esophageal Cancer: From Staging to Strategy—A Narrative Review

**DOI:** 10.3390/diagnostics15222867

**Published:** 2025-11-12

**Authors:** Francesca Lusetti, Roberta Muscia, Ermelinda D’Alessandro, Giuseppe Fierro, Gianpiero Manes, Germana de Nucci

**Affiliations:** 1 Gastroenterology Unit, Asst Rhodense, Garbagnate Milanese, 20024 Milan, Italy; flusetti@asst-rhodense.it (F.L.); gfierro@asst-rhodense.it (G.F.);; 2 Gastroenterology Unit, AOU Federico II University, 80131 Naples, Italy; ermelinda.d95@gmail.com

**Keywords:** squamous cell esophageal cancer, endoscopic ultrasound, EUS, esophagus

## Abstract

Esophageal squamous cell carcinoma (ESCC) remains a major global health challenge due to its aggressive nature and frequent late-stage diagnosis. Accurate locoregional staging is critical for guiding appropriate therapy, and endoscopic ultrasound (EUS) has emerged as the preferred modality for assessing tumor depth and regional lymph node involvement. In this narrative review, we provide a comprehensive overview of the role of EUS in the management of ESCC, from initial staging to post-treatment assessment. We discuss its strengths and limitations, particularly in differentiating early-stage disease and in restaging after neoadjuvant therapy. The importance of a multimodal approach—integrating EUS with computed tomography (CT), positron emission tomography (PET), and histologic sampling—is emphasized to improve diagnostic precision. We also explore emerging techniques, such as contrast-enhanced EUS, elastography, and novel therapeutic strategies including immune checkpoint inhibitors and endoscopic mucosal resurfacing. While EUS remains a cornerstone in the management of ESCC, ongoing innovation and integration with personalized medicine are expected to further enhance its clinical impact.

## 1. Introduction

Esophageal squamous cell carcinoma (ESCC) is a malignant epithelial neoplasm that arises from the stratified squamous mucosa of the esophagus. It is histologically and epidemiologically distinct from esophageal adenocarcinoma (EAC), which originates from glandular epithelium and is typically associated with Barrett’s esophagus and gastroesophageal reflux disease (GERD) [1,2,3]. ESCC predominantly involves the mid to upper esophagus and is more strongly linked to lifestyle-related exposures such as tobacco use, alcohol consumption, poor nutritional status, and ingestion of hot or nitrosamine-rich foods [4,5,6,7,8]. It remains the dominant histologic subtype of esophageal cancer globally, especially in regions such as Eastern Asia, South-Central Asia, and parts of sub-Saharan Africa [9,10].

Despite improvements in oncologic management, esophageal cancer remains a major cause of cancer-related mortality, ranking as the 11th most common cancer and the 7th leading cause of cancer death worldwide [10]. In 2020, an estimated 604,100 new cases and 544,100 deaths were reported, with ESCC accounting for approximately 85% of all esophageal cancers [1]. Prognosis remains poor: the five-year survival rate for esophageal cancer typically ranges from 10% to 30%, depending on stage at diagnosis and access to care. Projections suggest that, if current trends persist, the global burden of esophageal cancer will rise to nearly 957,000 new cases and 880,000 deaths annually by 2040, with ESCC expected to remain the dominant subtype [1].

The aggressive biology of ESCC, characterized by early submucosal invasion and a high rate of lymphovascular spread, demands accurate locoregional staging to guide therapeutic planning. While computed tomography (CT) and positron emission tomography (PET-CT) are useful for assessing distant metastases, they are limited in evaluating tumor depth and periesophageal lymph nodes.

**Figure 1:**
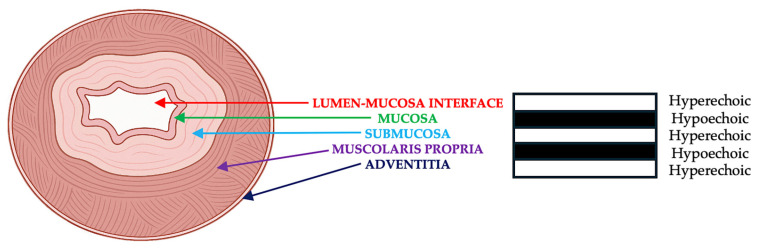
Anatomical cross-section of the esophageal wall showing its five-layer structure, as visualized by EUS. The layers include: 1—the interface between the lumen and mucosa (hyperechoic), 2—the deep mucosa (hypoechoic), 3—the submucosa (hyperechoic), 4—the muscularis propria (hypoechoic), 5—the adventitia (hyperechoic). This echogenic pattern allows for differentiation of tumor invasion depth, facilitating accurate T staging in patients with esophageal squamous cell carcinoma.

Endoscopic ultrasound (EUS) has become the modality of choice for locoregional staging in this setting. EUS is a minimally invasive imaging technique that combines endoscopy with high-frequency ultrasonography to provide high-resolution cross-sectional imaging of the esophageal wall and adjacent structures [11,12]. It enables precise visualization of the five-layer structure of the esophageal wall (Figure 1), allowing for accurate T staging and the detection of regional lymph node metastases [13,14].

Importantly, the addition of EUS-guided fine-needle tissue sampling—either aspiration (EUS-FNA) or biopsy (EUS-FNB)—enables cytological or histological confirmation of suspicious lymph nodes, thereby improving the accuracy of nodal staging and refining treatment algorithms.EUS also plays an integral role in selecting candidates for minimally invasive therapies such as endoscopic mucosal resection (EMR) and endoscopic submucosal dissection (ESD), which may offer curative treatment in early-stage disease [14,15]. Moreover, it supports response assessment following neoadjuvant chemoradiotherapy and is being explored for therapeutic applications including fiducial placement, injection therapy, and EUS-guided drainage or ablation in select cases [13].

Despite its strengths, EUS is not without limitations. Tumor-related factors such as luminal stenosis can preclude complete examination, and post-treatment fibrosis often obscures tissue planes and impairs interpretation—especially after neoadjuvant therapy [13,14]. Operator experience further modulates diagnostic performance; several studies report substantial interobserver variability in both T- and N-staging, particularly in low-volume centers [13,14]. Emerging therapeutic advances—including immunotherapy and molecular stratification—are already reshaping treatment algorithms and require clinicians to re-evaluate the role of EUS in patient selection and monitoring [16].

The aim of this narrative review is to provide a comprehensive overview of the current and emerging roles of endoscopic ultrasound in the management of esophageal squamous cell carcinoma, encompassing its application from diagnosis and staging to therapeutic decision-making and response assessment.

## 2. Methods

We conducted a narrative review on the role of EUS in squamous cell esophageal cancer. A comprehensive search was performed in PubMed, Embase, and Scopus from inception to 31 August 2025. The following search terms and their combinations were used: endoscopic ultrasound, EUS, esophageal cancer, esophageal neoplasms, squamous cell carcinoma, staging, diagnosis, treatment planning. Major international guidelines were also screened. Relevant abstracts from recent gastroenterology and oncology congresses were considered. Two authors (FL and GDN) independently reviewed all retrieved articles and reference lists to identify additional pertinent studies.

## 3. Relevant Sections

### 3.1. EUS for Locoregional Staging in Esophageal Squamous Cell Carcinoma

EUS, combining endoscopy with high-frequency ultrasonography, allows detailed visualization of the gastrointestinal wall and adjacent structures. In the context of ESCC, EUS has become an essential tool for locoregional staging and therapeutic decision-making [17,18,19,20]. Its ability to delineate the individual layers of the esophageal wall makes it particularly suitable for assessing tumor invasion depth, while its proximity to periesophageal lymphatic stations facilitates the detection of regional nodal metastases.

Compared with cross-sectional imaging modalities such as computed tomography (CT) and positron emission tomography (PET), EUS provides superior spatial resolution in the assessment of the esophageal wall and adjacent lymph nodes [21,22,23]. As a result, it plays a central role not only in initial staging, but also in the selection of candidates for endoscopic therapies, in guiding tissue acquisition through fine-needle aspiration (EUS-FNA), and in post-treatment response assessment.

A clear understanding of the TNM classification system is fundamental for interpreting EUS-based staging findings. The American Joint Committee on Cancer (AJCC) and Union for International Cancer Control (UICC) 8th edition TNM definitions for esophageal cancer are summarized in Table 1 [24].

#### 3.1.1. T-Staging with EUS

The capacity of EUS to distinguish the five echolayers of the esophageal wall enables accurate determination of tumor penetration. EUS is particularly recommended in patients who are candidates for curative treatment. Its ability to assess both the depth of tumor invasion and regional lymph node involvement makes it especially valuable in cases with suspected T1b lesions or beyond [25].

In a recent systematic review Inoue et al. reported a diagnostic accuracy of 83.3% for T-staging in patients with ESCC, highlighting substantial agreement with postoperative pathological findings (κ = 0.76), particularly for T1 and T3 tumors [26]. Comparable accuracy figures had already been suggested in earlier analyses, including the historical meta-analysis by Puli et al., which reported pooled sensitivities of 81.6% for T1, 81.4% for T2, 91.4% for T3, and 92.4% for T4, with specificities exceeding 94% across stages [27]. However, differentiation between T1a and T1b lesions remains challenging, with reported accuracy ranging from 70% to 85% due to limitations in resolving the interface between the muscularis mucosae and submucosa [28]. This limitation was further confirmed by He et al. in a retrospective study of patients with early-stage ESCC, where EUS achieved an overall accuracy of 70.8% and a sensitivity of 74.3% in differentiating T1a from T1b. Lesion length emerged as a significant determinant of diagnostic performance [29].

Beyond conventional EUS, new multimodal endoscopic strategies have been developed to overcome its intrinsic limitations. In a 2022 meta-analysis, Su et al. evaluated the combination of magnifying endoscopy with narrow-band imaging (ME-NBI) and EUS, reporting pooled sensitivity and specificity of 94.7% and 89.4% for detecting early esophageal cancer, and 79.1% and 94.3% for accurately staging invasion depth (AUC = 0.95). These findings suggest that integrating high-resolution endoscopic imaging with EUS could enhance differentiation between mucosal and submucosal disease, although validation in larger, multiethnic cohorts is still required [30].

#### 3.1.2. N-Staging with EUS

Accurate nodal staging is essential in the management of ESCC, as lymphatic dissemination is common even in early-stage disease and significantly impacts therapeutic decisions and prognosis. EUS is the most sensitive modality for detecting periesophageal lymph node involvement, given its proximity and high-resolution imaging capabilities [13,17]. According to Inoue’s study, the overall accuracy of EUS for N-staging was 78.8%, with a κ coefficient of 0.59, indicating moderate agreement with pathological nodal status. The accuracy was particularly higher in patients with ≥T2 tumors, reaching up to 88.9% [26]. Already in 2008, using technology now considered outdated, Puli et al. reported a pooled sensitivity and specificity of 85% and 86%, respectively, for EUS in N-staging of esophageal cancer, demonstrating its superiority over CT and PET in detecting locoregional nodal metastases [27]. The diagnostic yield of EUS increases further with the addition of fine-needle or fine-needle biopsy sampling (EUS-FNA/FNB), which allows cytological or histological confirmation of suspicious nodes and reduces false positives due to reactive changes [17]. EUS-TA should be performed for any morphologically suspicious lymph node, as recommended by the ESGE. Typical targets include round, hypoechoic nodes with sharp margins and a short-axis diameter > 5 mm. A 22-gauge (G) needle is generally preferred, while 25 G may be used in angulated positions and 19 G when a core sample is required. At least two to three passes are advised, using slow-pull or capillary suction techniques, and on-site cytopathology (ROSE) can further increase adequacy [31].

The diagnostic performance of EUS-guided tissue sampling for lymph-node assessment has been investigated in several studies, though data remain heterogeneous across populations and target sites. In a meta-analysis by Tamanini et al. (2021) including 26 studies and 2833 lymph nodes, the pooled sensitivity was 87% and specificity 100%, with an area under the curve (AUC) of 0.99 and a low adverse-event rate (~1.6%) [32]. These figures reflect the overall accuracy of EUS-guided tissue acquisition, encompassing both fine-needle aspiration (FNA) and fine-needle biopsy (FNB) techniques. The authors noted that most studies included in the analysis used FNA needles, whereas for solid lesions—such as pancreatic masses—FNB is generally preferred to obtain core tissue [33]. This likely reflects the fact that lymph nodes are typically less fibrotic and more cellular, allowing adequate cytologic yield even with FNA [32]. Current ESGE guidelines do not favor one approach over the other, as both techniques achieve comparable diagnostic accuracy when performed by experienced operators [31,34].

### 3.2. Operator Dependence and Institutional Volume

While EUS-guided nodal staging outperforms CT or PET-CT, its accuracy is tightly linked to endoscopist expertise and center volume. In the multicentre study by van Vliet et al., overall T + N accuracy was 56% in a unit performing < 50 EUS/year versus 79–83% in high-volume units (>200/year), leading to a 23% stage-migration rate that directly altered treatment allocation and adherence to NCCN/ESMO algorithms [35]. A similar two-fold rise in staging errors occurred when examinations were performed by non-expert operators in the series of Lee et al. [20]. These data underpin current society recommendations for competency-based training (≈150 supervised cases with periodic audit) and minimum-volume thresholds for centers offering curative-intent staging [36,37,38].

Beyond institutional volume, the operator’s training pathway is a crucial determinant of diagnostic reliability. Structured EUS education, combining progressive supervision with standardized assessment, has become the cornerstone of competency development. According to the European Society of Gastrointestinal Endoscopy (ESGE) and United European Gastroenterology (UEG) curriculum, trainees should complete at least 225 supervised diagnostic procedures and 50 EUS-FNA/FNB before independent practice [39]. The curriculum also sets quality benchmarks for trainees, recommending that at least 85% of tissue samples obtained by EUS-FNA/FNB should be adequate for diagnosis, and that the overall diagnostic accuracy should exceed 90% for solid lesions, reflecting the thresholds associated with optimal clinical outcomes [39].

To achieve these standards, simulation-based training—including practice on phantoms, animal models, and increasingly on virtual-reality platforms—is encouraged in the early stages to develop both cognitive and technical proficiency. Nevertheless, the implementation of such structured programs remains inconsistent across Europe, and access to high-fidelity simulators or mentored case exposure is often limited. Consequently, many centres still rely on experiential, apprenticeship-style learning, which contributes to interoperator variability.

In low-volume institutions, additional strategies such as hub-and-spoke referral networks, tele-mentoring, and centralized certification may help ensure consistent quality. Continuous audit and periodic recertification, as promoted by ESGE and UEG, are essential to maintain long-term competence and reproducibility of EUS-based staging.

### 3.3. Multimodal Staging Approach: Combining EUS, CT, and PET-CT

To achieve accurate staging and guide appropriate therapeutic planning, current international guidelines recommend a multimodal approach combining contrast-enhanced computed tomography (CT), positron emission tomography integrated with CT (PET-CT), and endoscopic ultrasound (EUS), the latter of which has already been discussed in detail in the previous section [36,37,38,40,41,42].

- Contrast-enhanced CT remains the initial imaging tool for most patients with suspected esophageal cancer. It provides essential anatomical information, including tumor length, local invasion into adjacent structures, presence of enlarged lymph nodes, and detection of distant metastases—particularly in the lungs, liver, and adrenal glands [36,40]. CT also plays a central role in evaluating resectability by identifying invasion of unresectable structures (e.g., aorta, trachea) and assessing celiac or supraclavicular nodal involvement. However, its sensitivity for early wall invasion (T1–T2) and small-volume nodal disease is limited, especially in the absence of size enlargement [26].
- PET-CT, using [18F]-fluorodeoxyglucose, complements CT by providing metabolic imaging that can detect distant metastases with higher sensitivity, especially in sites that may appear morphologically normal on CT, such as bone, lymph nodes, or liver. PET-CT is particularly valuable for identifying occult systemic disease, thereby preventing unnecessary surgery or inappropriate local treatment [36,43]. Moreover, it has prognostic implications, as higher standardized uptake values (SUV) have been associated with more aggressive disease biology and worse outcomes. Importantly, PET alone is rarely used in current practice, while it is almost always integrated with CT (PET-CT) to combine metabolic and anatomical information. Some guidelines specifically recommend contrast-enhanced PET-CT, though availability may vary by institution [40,41]. While PET-CT is widely used, it is not necessarily indicated for all patients. According to several guidelines, its use is generally recommended in patients considered for curative treatment, especially surgical or combined-modality approaches, to rule out distant disease. It may be omitted in frail patients or those with clearly unresectable tumors on CT alone [36,38].

Together, CT and PET-CT complement EUS by covering its limitations: while EUS is superior for assessing local invasion and regional nodes, CT and PET-CT are essential for mapping distant spread and surgical planning. Thus, all three modalities are often integrated in a stepwise or parallel fashion based on the clinical context.

### 3.4. Treatment Strategies for ESCC According to Disease Stage

**Figure 2:**
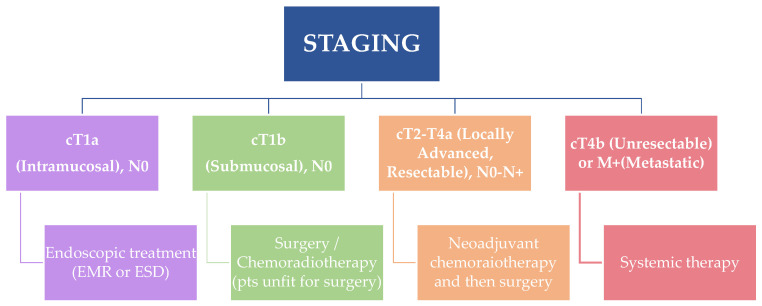
Flowchart of treatment strategies according to different disease stage.

Management of ESCC is primarily guided by clinical staging and patient fitness, with therapeutic approaches ranging from endoscopic resection in early-stage disease to multimodal strategies for locally advanced or metastatic cases (Figure 2). The use of a multimodal diagnostic pathway, as previously discussed, ensures accurate staging and appropriate treatment allocation.

- cT1a (Intramucosal)–N0

For patients with cT1a ESCC, endoscopic treatment is the standard of care. Endoscopic mucosal resection (EMR) or endoscopic submucosal dissection (ESD) are considered curative if R0 resection is achieved and there is no lymphovascular invasion or poor differentiation [15,38,40,41,42]. ESD is generally considered superior to EMR in achieving en bloc resection and minimizing local recurrence rates [44]. ESD is generally favored over EMR for lesions larger than 15 mm or when there is suspicion of deeper invasion, due to its higher rates of en bloc and R0 resection, as well as lower local recurrence [25]. A recent pilot randomized study with 5-year follow-up evaluated the use of background mucosal resurfacing after curative ESD and found it significantly reduced rates of metachronous recurrence, suggesting a promising preventive strategy in high-risk patients [45].

- cT1b (Submucosal)–N0

Submucosal invasion significantly increases the risk of lymph node metastasis. Therefore, surgical esophagectomy with lymphadenectomy is generally recommended. Minimally invasive esophagectomy (MIE) has gained ground as a surgical option for early-stage ESCC, offering reduced operative trauma, faster recovery, and fewer postoperative complications compared to traditional open surgery. Nonetheless, its broader implementation is challenged by the need for advanced skills and specialized equipment [46]. In Eastern countries, particularly Japan, extended lymphadenectomy with three-field dissection is more frequently adopted in the surgical management of ESCC, based on evidence suggesting a survival advantage. This contrasts with Western practice, where two-field dissection remains the standard technique [46].

For patients unfit for surgery or those refusing it, chemoradiotherapy may be considered as an alternative definitive treatment.

- cT2–T3, N0–N+ (Locally Advanced, Resectable)

The standard approach for cT2–T3 tumors or node-positive disease is neoadjuvant chemoradiotherapy (nCRT) followed by surgery. The CROSS trial demonstrated improved overall survival with this approach, and it is endorsed by ESMO and NCCN guidelines [36,38,47]. The Japanese guidelines prefer neoadjuvant chemotherapy over chemoradiotherapy for this setting, commonly using cisplatin and 5-FU [40,41].

- cT4a (Resectable)

For tumors invading resectable adjacent structures (pleura, pericardium, diaphragm), treatment strategies align with those for locally advanced ESCC, involving neoadjuvant therapy followed by surgery when technically feasible.

- cT4b (Unresectable) or M1 (Metastatic)

Systemic therapy is the mainstay for unresectable or metastatic ESCC. First-line systemic treatment depends on the expression of programmed death-ligand 1 (PD-L1). For patients with a PD-L1 combined positive score (CPS) ≥ 10, immunotherapy with nivolumab plus chemotherapy (e.g., 5-FU and cisplatin) is the preferred option, based on data from the CheckMate 648 trial [48].

Patients with lower CPS or contraindications to immunotherapy may receive doublet chemotherapy. Supportive and palliative care should be integrated early in the disease course to maintain quality of life [36,37,38].

**Figure 3:**
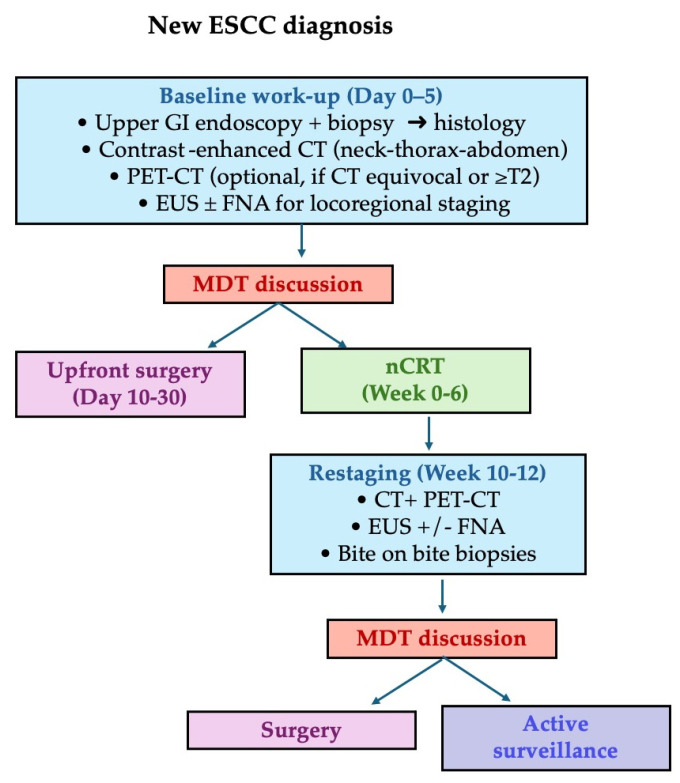
Proposed algorithm for the diagnostic-therapeutic work-up in ESCC. Abbreviations: CT = computed tomography; PET-CT = positron-emission tomography–CT; EUS = endoscopic ultrasound; FNA = fine-needle aspiration; MDT = multidisciplinary tumor board; nCRT = neoadjuvant chemoradiotherapy.

A summary flowchart of the recommended staging pathway is provided in Figure 3.

### 3.5. EUS for Restaging and the Challenge of Understaging

Restaging patients with esophageal squamous cell carcinoma (ESCC) after neoadjuvant chemoradiotherapy (nCRT), as shown in Table 2, remains challenging. Although endoscopic ultrasound (EUS) is indispensable for initial T- and N-staging, its post-treatment performance is markedly reduced by fibrosis, inflammation, and disrupted tissue planes. In a retrospective series by de Nucci et al. (2021), EUS correctly staged only 45% of tumors after nCRT, with most apparent “residual” lesions later proven to be complete responders on histology. Such overstaging highlights the difficulty of distinguishing residual malignancy from post-therapeutic changes [49].

Similarly, Eyck et al. (2020) confirmed the limitations of EUS after nCRT: their meta-analysis showed high sensitivity for residual primary tumour (96%) but extremely low specificity (8%), generating many false positives. For nodal restaging they recorded only modest performance, with pooled sensitivity and specificity of 68% and 57%, respectively [50].

These findings are consistent with those reported by van der Bogt et al. (2019), who found that EUS alone identified only 50% of residual malignant nodes post-nCRT [51]. Yet, even with EUS-FNA, small malignant nodes (<5 mm) were often missed, as previously emphasized by van Vliet et al., illustrating the limitations of morphology-based criteria [35,51].

Given these limitations, EUS-derived quantitative parameters—such as residual tumor thickness and cross-sectional area measured 12 weeks post-nCRT in the PreSANO trial subanalysis—achieved sensitivities of 87–89% but specificities of only 40–52% [52]. Emerging techniques—such as contrast-enhanced EUS and elastography—have shown promise in preliminary studies, but their clinical validity must still be confirmed through prospective trials [25].

Therefore, no single modality provides sufficient accuracy in the post-nCRT setting. Integrated restaging—combining EUS (with high-resolution imaging and FNA), PET-CT, targeted endoscopic biopsies, and, when indicated, cross-sectional imaging—should be interpreted by a multidisciplinary team. PET-CT, for instance, yields roughly 74% sensitivity and 52% specificity on qualitative reads, marginally improved by metabolic response criteria (SUVmax reduction) to 73–63% [50,51].

Clinicians routinely perform endoscopic biopsies after nCRT, yet sensitivity remains sub-optimal [51]. According to van der Wilk et al., standard biopsies detect only 15% of residual tumors, while bite-on-bite biopsies modestly improve sensitivity to 34% [53]. Despite high specificity, their limited negative predictive value underscores the need for complementary modalities in restaging.

In clinical practice, restaging decisions should be guided by converging findings across modalities rather than reliance on EUS alone. A cautious, multimodal strategy—under the oversight of surgeons, medical and radiation oncologists, radiologists, and pathologists—maximizes accuracy and informs optimal post-treatment management.

## 4. Discussion

In this narrative review, we examine the role of EUS as a cornerstone of ESCC management, from precise locoregional staging to guiding therapy and monitoring response. By visualizing esophageal wall layers and regional nodes with submillimetric detail, EUS provides unparalleled anatomic definition and remains the most accurate technique for local staging. Nevertheless, the modality is not without limitations: distinguishing T1a from T1b lesions and identifying lymph nodes < 1 cm continue to represent diagnostic blind spots, with sensitivity reported between 70 and 85% and marked operator variability [28,29].

In a multicentre ESCC cohort, Kahlon et al. reported 82% sensitivity and 77% specificity for T1a/T1b staging, but also documented substantial inter-centre variability [54]. To address these limitations, several methodological refinements have been proposed. Zhang et al. demonstrated that adding a submucosal saline injection during EUS improved T1a/T1b accuracy from 78% to 92% in superficial ESCC [55], while other studies have explored the integration of magnifying endoscopy or narrow-band imaging to enhance mucosal layer definition [30].

Following neoadjuvant therapy, however, treatment-induced fibrosis and inflammation further compromise accuracy, reinforcing the need to integrate EUS with complementary imaging for comprehensive evaluation [49,50]. Consequently, contemporary guidelines advocate combining EUS with CT and PET-CT for comprehensive anatomical and metabolic evaluation [36].

However, whether EUS should be routinely performed after PET/CT in all patients remains controversial. According to current international guidelines, EUS is strongly recommended for patients who are potential candidates for curative treatment, as it refines T and N staging and informs therapeutic decision-making. Nevertheless, both the NCCN and ESMO guidelines acknowledge that EUS may be omitted when PET/CT or CT has already demonstrated distant metastases, or when severe luminal stenosis precludes safe passage of the endoscope [36,37,38]. Similarly, the Japan Esophageal Society guidelines propose a stepwise diagnostic strategy, reserving EUS for cases in which locoregional staging will impact treatment selection, and omitting it in clearly unresectable or metastatic disease [40,41]. Therefore, the indication for EUS should be individualized, balancing its diagnostic yield against procedural feasibility and expected clinical benefit. EUS adds limited value in patients with clearly unresectable, metastatic, or circumferentially obstructing tumors, where the information obtained is unlikely to modify therapeutic strategy.

In this context, accurate EUS-based staging is fundamental for appropriate treatment allocation, helping distinguish candidates for endoscopic resection, multimodal therapy, or systemic management. Endoscopic resection (preferably ESD) is indicated for selected early tumors, while nCRT followed by surgery remains the standard for locally advanced disease in Western settings. Definitive chemoradiation is often preferred in some Eastern centers, whereas systemic therapy is reserved for unresectable cases—each strategy balancing oncologic efficacy and patient safety.

Beyond diagnostic performance, cost-effectiveness and resource allocation must also be considered. Advanced EUS techniques, such as elastography and contrast-enhanced modalities, require specialized equipment and expertise, which may not be equally available across all healthcare systems. In resource-limited settings, the widespread adoption of such technologies may be challenging, underscoring the need to balance diagnostic precision with accessibility and cost sustainability. Ultimately, the role of EUS in ESCC will depend not only on technological innovation but also on equitable training, standardization, and validation of its true clinical impact.

## 5. Future Directions

The clinical role of EUS in ESCC is likely to broaden considerably over the next decade, supported by both technological progress and evolving oncologic strategies. Among the innovations closest to clinical integration are contrast-enhanced EUS (CE-EUS) and EUS elastography, which improve visualization of vascularity and tissue stiffness, enhancing the assessment of tumor depth and nodal involvement. These modalities are already established in other gastrointestinal cancers and are being progressively adopted in ESCC staging, though large-scale prospective validation is still ongoing [13,19].

By contrast, artificial intelligence (AI) in endoscopic imaging remains in the experimental phase. Initial studies have shown that AI algorithms can analyze EUS and endoscopic images with accuracy comparable to expert endosonographers. In the study by Huang et al., deep-learning models trained on gastrointestinal EUS—including ESCC—accurately identified malignant features and lymph node metastases [56]. Similarly, Zhang et al. (2024) developed a ResNet-152 convolutional neural network capable of predicting tumor depth using white-light endoscopy images, reporting AUC values between 0.94 and 0.98 [57], while the Interpretable Depth Prediction System (AI-IDPS) applied magnifying endoscopy with narrow-band imaging (ME-NBI) to accurately distinguish superficial from deeply invasive lesions, with accuracies above 85% [58].

Although encouraging, current evidence is limited to single-centre, retrospective studies, and prospective multicentre validation is required before AI can be applied in routine ESCC staging. Regulatory approval, technical standardization, and adequate training remain major barriers, alongside medico-legal and data privacy concerns that must be resolved to enable safe clinical implementation [59].

Emerging therapeutic adjuncts are also under active investigation. In a randomized controlled pilot trial, Wang et al. demonstrated that background mucosal resurfacing (BMR) following ESD markedly reduced metachronous recurrence (0% vs. 53% at five years, *p* = 0.001), highlighting a potentially valuable preventive strategy for early ESCC [45].

Finally, molecular and immunologic profiling is reshaping ESCC management and progressing at different stages of clinical translation [46,60,61]. Immune checkpoint inhibitors targeting the PD-1/PD-L1 axis have already entered clinical practice, demonstrating improved survival both in neoadjuvant protocols—where they enhance pathological response rates—and in advanced disease, where they prolong overall survival and are now part of standard systemic therapy [62]. In contrast, molecularly targeted therapies (e.g., EGFR, VEGFR, and FGFR inhibitors) and the incorporation of circulating tumor DNA (ctDNA) or next-generation sequencing remain investigational approaches, currently limited to clinical trials [62].

Collectively, these developments highlight the evolution of EUS from a staging tool to an integrated platform that combines validated imaging advances with emerging technologies such as AI and molecular profiling. The next step will be to confirm their clinical value through prospective validation and standardized implementation.

## 6. Conclusions

In conclusion, this narrative review emphasizes the central role of EUS in the multidisciplinary management of ESCC, particularly for accurate locoregional staging and treatment stratification. Despite inherent limitations—especially in early-stage disease and post-treatment settings—EUS remains superior to cross-sectional imaging in assessing tumor infiltration and regional lymph node involvement. Ongoing innovations, such as enhanced imaging technologies and the integration of molecular and immunologic therapies, are expected to further refine patient selection and optimize therapeutic outcomes.

## Figures and Tables

**Table 1:** TNM Classification for Esophageal Cancer (AJCC 8th Edition) [24].

Category	Definition
**T1A**	Tumor invades lamina propria or muscularis mucosae
**T1B**	Tumor invades submucosa
**T2**	Tumor invades muscularis propria
**T3**	Tumor invades adventitia
**T4A**	Tumor invades pleura, pericardium, diaphragm, or adjacent resectable structures
**T4B**	Tumor invades aorta, vertebral body, trachea, or other unresectable structures
**N0**	No regional lymph node metastasis
**N1**	Metastases in 1–2 regional lymph nodes
**N2**	Metastases in 3–6 regional lymph nodes
**N3**	Metastases in 7 or more regional lymph nodes
**M0**	No distant metastasis
**M1**	Distant metastasis present

**Table 2:** Comparative sensitivity and specificity of EUS, PET-CT, and biopsy for restaging after neoadjuvant chemoradiotherapy (nCRT) in ESCC.

Modality	Sensitivity	Specificity	Key Limitations
EUS	96% (primary tumor) [44]; 68% (nodes) [44]	8% (primary tumor) [44]; 57% (nodes) [44]	Overstaging due to fibrosis and inflammation
PET-CT	~74% [44]	~52% [44]	Limited discrimination between viable tumor and inflammation; false positives
Biopsy post-nCRT	15–34% sensitivity [47]	~100% [47]	Low negative predictive value; sampling error

Abbreviations: EUS = endoscopic ultrasound; PET-CT = positron emission tomography–computed tomography; nCRT = neoadjuvant chemoradiotherapy.

## Data Availability

The data presented in this study are available in Pubmed as the references list below.
